# Water in a nitrous oxide flowmeter

**DOI:** 10.4103/0019-5049.63633

**Published:** 2010

**Authors:** Virendra K Arya, Vikramjeet Arora

**Affiliations:** Postgraduate Institute of Medical Education and Research, Chandigarh - 160 012, India

**Figure 1:**
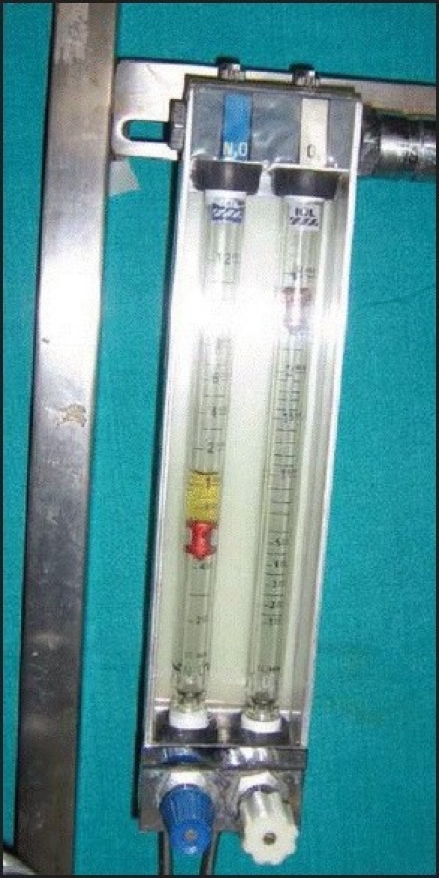
Nitrous oxide flowmeter control valve showing the `stuck bobbin´nd water level on top of the bobbin

**Figure 2:**
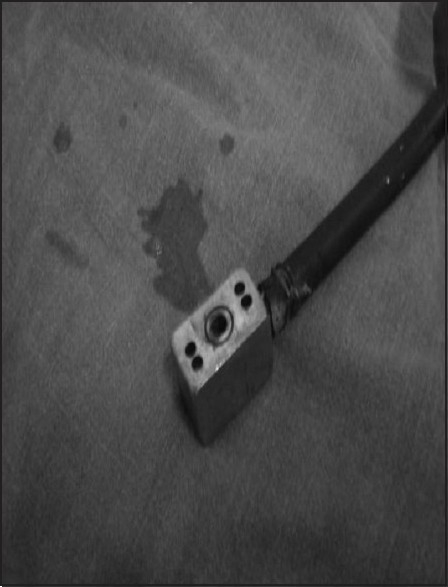
Water emerging from the yoke adapter of the nitrous oxide hose

Sir,

We are reporting a rare but critical incident wherein water was detected in a nitrous oxide (N_2_O) flowmeter. This incident occurred in the gynecology OR where the anaesthesiologist used an O_2_ / N_2_O mixture with a volatile agent for maintenance of anaesthesia. After about one to two minutes of opening the N_2_ O flowmeter control valve, a spurt of yellowish colored fluid from the needle valve filled the flowmeter barrel and the bobbin jumped erratically up and down on it. The fluid got accumulated over the bobbin making it stick to the side of the barrel and cease its rotations [Figure 1]. The total flow of the gas from the common gas outlet also decreased, as evidenced by the empty feel of the ventilating reservoir bag. The N_2_O was immediately shut off and patient was maintained on 100% O_2_ from the cylinder supply, while ventilating with a self-inflating resuscitation bag. Fortunately, the fluid did not travel distally into the breathing system. On disconnection of the N_2_O hose from the terminal unit and connecting yoke, some water was found emerging from the yoke adapter [Figure 2]. On purging the terminal units no contamination of gases with any fluid was observed. No problem was encountered after using new hoses and a new anaesthesia machine.

Subsequent investigation revealed that on the previous day gas hoses of the same OR were disconnected for maintenance. Subsequently, only the O_2_hose was connected by the maintenance staff and the N_2_O hose remained disconnected from the terminal unit overnight. It was possible that the water vapour entering from the atmosphere could have condensed under pressure on reconnection in the morning. A routine morning check of the machine did not reveal any problem due to the small quantity of water, which took some time to reach up to the flowmeter. A similar incident had been reported earlier, where a central air pipeline was found contaminated by water condensation, as it was kept open to atmosphere during maintenance.[1]We feel that purging of hoses must be done with central pipeline dry gases before connecting the yoke adapter to the machine for prevention of this problem. In addition to patient safety concerns, any water in the gas supply would also lead to malfunction of vaporisers and a major break down in the modern anaesthesia machines with electronic components.
